# Identification of a new pseudogenes/lncRNAs-hsa-miR-26b-5p-COL12A1 competing endogenous RNA network associated with prognosis of pancreatic cancer using bioinformatics analysis

**DOI:** 10.18632/aging.103709

**Published:** 2020-10-07

**Authors:** Shilei Jing, Jiao Tian, Yanpeng Zhang, Xinhua Chen, Shusen Zheng

**Affiliations:** 1 Department of Surgery, Division of Hepatobiliary and Pancreatic Surgery, First Affiliated Hospital, School of Medicine, Zhejiang University, Hangzhou 310000, China; 2 NHFPC Key Laboratory of Combined Multi-Organ Transplantation, Hangzhou 310000, China; 3 Department of Respiratory, Tianjin Children's Hospital, Tianjin 300134, China; 4 Key Laboratory of the Diagnosis and Treatment of Organ Transplantation, CAMS, Hangzhou 310000, China; 5 Key Laboratory of Organ Transplantation, Zhejiang Province, Hangzhou 310003, China; 6 Collaborative Innovation Center for Diagnosis Treatment of Infectious Diseases, Hangzhou 310000, China

**Keywords:** pancreatic cancer, miRNA, pseudogene, lncRNA, COL12A1

## Abstract

Background: Pancreatic carcinoma is one of the most malignant cancers globally. However, a systematic mRNA-miRNA-lncRNA/pseudogene network associated with the molecular mechanism of pancreatic cancer progression has not been described.

Results: The significant DEGs identified comprised 159 up-regulated and 92 down-regulated genes. According to the expression and survival analysis, three genes (COL12A1, APOL1, and MMP14) were significantly higher in tumor samples when compared with normal controls and their upregulation indicated a poor prognosis. Subsequently, 28, 17, and 11 miRNAs were predicted to target COL12A1, APOL1, and MMP14, respectively. The hsa-miR-26b-5p-COL12A1 axis showed a potential in suppressing the progression of pancreatic cancer. Moreover, 12 lncRNAs and 92 pseudogenes were predicted to potentially bind to the hsa-miR-26b-5p. Based on the results from expression and correlation analysis, NAMPTP1/HCG11-hsa-miR-26b-5p-COL12A1 competing endogenous RNA (ceRNA) sub-network was associated with the prognosis of pancreatic cancer.

Conclusions: In a word, we elucidate a new NAMPTP1/ HCG11-hsa-miR-26b-5p-COL12A sub-network in the progression of pancreatic cancer, which may serve as a promising diagnostic biomarker or effective therapeutic target for pancreatic cancer.

Materials and methods: Differentially expressed genes (DEGs) were first identified by mining GSE28735, GSE62452 and GSE41368 datasets. Functional enrichment analysis was conducted using the DAVID database. Protein-protein interaction (PPI) network was performed using the STRING database, and hub genes were identified by Cytoscape. Upstream miRNAs and pseudogenes /lncRNAs of mRNAs were forecast using miRTarBase, miRNet, and starBase. Expression, survival, and correlation analysis of genes, miRNAs, and pseudogenes /lncRNAs were validated using GEPIA, Kaplan-Meier, and starBase.

## INTRODUCTION

Pancreatic carcinoma is considered as one of the most malignant and the fourth leading form of cancer mortality globally. The median survival time of pancreatic carcinoma is about 3-6 months and the 5-year survival rate is 9.1%, much less than that of other cancers [1, 2]. Pancreatic ductal adenocarcinoma (PDAC) accounts for approximately 90% of all pancreatic cancer cases [3]. Currently, therapeutic means including surgical resection, chemotherapy, and molecular targeted therapy effectively improve the prognosis in early-stage PDAC, but are not appropriate for advanced cases especially those involving systemic metastasis. Therefore, a comprehensive understanding of the molecular mechanisms of pancreatic cancer and the development of effective early diagnostic and therapeutic strategies are urgently required.

Most of the genome can be transcribed into RNAs consist of coding RNAs (mRNAs) and noncoding RNAs (ncRNAs) [4]. Approximately 98–99% of RNAs belong to noncoding RNA (ncRNA), including microRNA (miRNA), long ncRNA (lncRNA), pseudogene, and circular RNA (circRNA); which have key implications for human cancer [5–7]. In 2011, Salmena et al. proposed the competitive endogenous RNA (ceRNA) theory, which plays a crucial regulatory role between mRNAs and ncRNAs [8]. Since then, Increasing lncRNAs, pseudogenes, and circRNAs have been found and act as ceRNAs binding to miRNAs, thereby affecting disease progression [9–12]. However, there is an extremely limited number of ncRNAs that have been characterized functionally in PDAC. Therefore, it is essential to explore the potential molecular mechanism and novel therapeutic targets of pancreatic cancer from the perspective of ceRNA.

In this study, we constructed a network associated with the progression of pancreatic cancer through a series of bioinformatics analyses. First, we acquired differentially expressed genes (DEGs) by mining three GEO datasets. Next, protein-protein interaction (PPI) analysis was employed, and hub genes were identified. Subsequently, the most potential miRNA (hsa-miR-26b-5p) binding to COL12A1 was identified based on expression correlation and survival analysis. Finally, the potential upstream dysregulated mechanisms (pseudogenes and lncRNAs) were further explored. To the best of our knowledge, this is the first bioinformatics study to introduce pseudogenes into the investigation of specific ceRNA network in pancreatic cancer. The established pseudogenes/lncRNAs-hsa-miR-26b-5p-COL12A1 ceRNA sub-network may help us to comprehensively understand the pathogenesis of PDAC and provide promising diagnostic biomarkers or effective therapeutic targets for pancreatic cancer.

## RESULTS

### Screening of significant DEGs in pancreatic carcinoma

**Figure 1:**
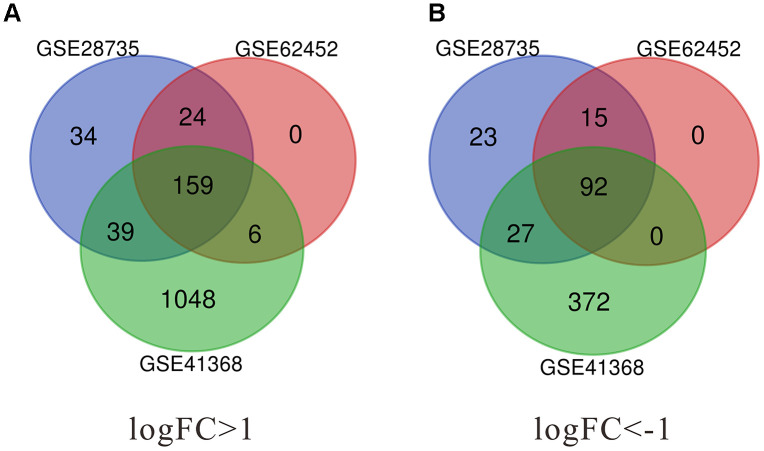
**Identification of significant differentially expressed genes (DEGs) in pancreatic cancer.** (**A**) The intersection of upregulated DEGs of GSE28735, GSE62452 and GSE41368 datasets. (**B**) The intersection of downregulated DEGs of GSE28735, GSE62452 and GSE41368 datasets. DEGs, Differentially expressed genes. |log2FC| >1 and adj. p-value < 0.05 were set as the cut-off criteria.

In the present study, a total of 296 DEGs were identified from GSE62452, 413 from GSE28735, and 1743 from GSE41368. The Venn diagram software was used to extract the common DEGs in the datasets. As shown in Figure 1 and Table 1, 251 common DEGs were detected, consisting of 159 up-regulated and 92 down-regulated genes.

**Table 1:** All 251 common differentially expressed genes (DEGs) were detected from three profile datasets, including 159 up-regulated genes and 92 down-regulated genes in the PDAC tissues compared to normal tissues.

**DEGs**	**Genes names**
UP-regulated	PTPRR PLAU MMP7 SERPINB5 HEPH CEACAM6 MET ITGA3 TGM2 COL1A1 ANLN GALNT5///GALNT5 DGKH DCBLD2 TRIM31 ADGRF1 FNDC1 TSPAN8 SLC6A6 EDNRA LTBP1 NRP2 COL1A2///COL1A2 MFAP5 EPHA4 CST1 PLA2R1 LIPH FN1 LY75-CD302///LY75 IFI27 ARNTL2 ASAP2 LAMB3 TNFAIP6 LEF1 MYOF ANO1 DPCR1 VSIG1 COL5A2 ANKRD22 MIA-RAB4B///MIA S100A14 DDX60 OSBPL3 TMPRSS4 ANTXR1 KYNU CAPG CCL20 ADAM9 MATN3 OLR1 NPR3 SLPI GPRC5A NOX4 NT5E CTSK SULF2 GPX2 IL1RAP ACSL5 HPGD APOL1 CDH11 AREG ITGA2 GREM1 SCEL FBN1 SLC6A14 BGN LAMA3 MMP1 IGFBP5 COL8A1 MMP12 SLC2A1 CD109 SLC22A3 DHRS9 ADAMTS12 LAMP5 ECT2 COL3A1 LMO7 EDIL3 ASPM FAP PLEK2 INPP4B ANXA10 TCN1 POSTN MMP14 PLAT ANXA8L1///ANXA8 ITGB6///ITGB6 NQO1 ADAM28 SRPX2 CYP2C18 IFI44L CEACAM5 CEMIP TMC5 CTSE OAS2 HK2 MUC17 GABRP TOP2A MICAL2 AEBP1 SYTL2 THBS2 BCAS1 GPX8 DSG3 RUNX2 KRT7 TSPAN1 KRT19 VCAN INHBA///INHBA SULF1 ITGA11 LAMC2 CXCL5 GCNT3 COL6A3 EGLN3 MUC13 COL10A1 LCN2 ACTA2 PLAC8 CEACAM1 COL11A1 AHNAK2 ETV1 P4HA1///P4HA1 FBXO32 TFF1 TGFBI CLDN18 FGD6 CP TMC7 FXYD3 MBOAT2 SEMA3C DPYSL3 COL12A1 CENPF UGT1A3///UGT1A1///UGT1A4///UGT1A9///UGT1A5///UGT1A6///UGT1A7///UGT1A8///UGT1A10 LC44A4
DOWN-regulated	BTG2 SPINK1 PRSS3P2 CPA1 CTNND2 RGN PDIA2 PM20D1 BACE1 OR8D4 CTRC NUCB2 IAPP HOMER2 PLA2G1B GSTA2 SLC16A12 CELA3B ERP27 KIAA1324 SLC43A1 GNMT ANKRD62 PNLIP///COL1A2 CELP CELA2B NR5A2 CELA3A ACADL IL22RA1 PKHD1 GRPR BEX1 CEL CTRB2///CTRB1 ANPEP SLC39A5 CFTR NRG4 REG1A F11 MT1G CPA2 LMO3 AQP12B///AQP12A BNIP3 ERO1B G6PC2 GUCA1C FGL1 TTN FAM24B-CUZD1///CUZD1 C5 EGF FAM3B AQP8 CTRL SYCN NRCAM SCGN PNLIPRP1 KLK1 PAK3 CLPS CHRM3 MIR217 SLC17A4 TMED6 SST ALB TRHDE PAIP2B///VPS36 CPB1 PRSS3 KCNJ16 SERPINI2 GRB14 DCDC2 GP2 GATM SLC4A4 F8 DPP10 AOX1 GPHA2 PDK4 CELA2A RBPJL EPHX2 PRSS1 PNLIPRP2

### Functional enrichment analysis for the significant DEGs

**Figure 2:**
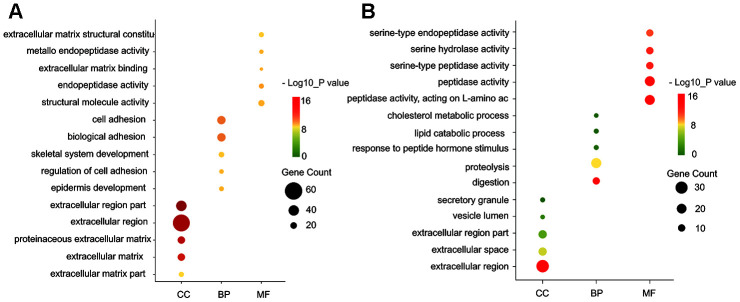
**GO functional annotation for the significant DEGs.** (**A**) The top five enriched cellular component (CC), biological process (BP) and molecular function (MF) of the upregulated significant DEGs. (**B**) The top five enriched cellular component (CC), biological process (BP) and molecular function (MF) of the downregulated significant DEGs. GO: Gene Ontology.

Gene ontology enrichment was performed using DAVID to further analyze the function of the DEGs. GO analysis results indicated that for biological processes (BP), the up-regulated DEGs were mainly enriched in cell adhesion, biological adhesion, blood vessel, and ectoderm development, while the down-regulated DEGs particularly in metabolic process, such as proteolysis, lipid catabolic process, and cholesterol metabolic process. For molecular function (MF), the up-regulated DEGs were enriched in extracellular matrix structural constituent, metallo-endopeptidase activity, extracellular matrix binding and calcium ion binding involved in cell-cell adhesion, while the down-regulated DEGs in peptidase activity, such as acting on L-amino acid peptides, serine-type peptidase activity and exopeptidase activity. For cell component (CC), the up-regulated DEGs were particularly enriched in extracellular matrix, fibrillar collagen, and plasma membrane, while the down-regulated DEGs in the extracellular region, vesicle lumen, secretory granule and cytoplasmic vesicle part (Figure 2A, 2B).

We also carried out DAVID to analyze the KEGG pathways enrichment. The analysis outcome revealed that included DEGs were significantly enriched in some well-known cancer-associated pathways including ECM-receptor interaction, focal adhesion, and PI3K-Akt signaling pathway (Table 2).

**Table 2:** KEGG pathway analysis of differentially expressed genes in pancreatic cancer.

**Pathway ID**	**Pathway description**	**Count**	**PValue**
hsa04974	Protein digestion and absorption	17	1.35E-12
hsa04972	Pancreatic secretion	17	3.30E-12
hsa04512	ECM-receptor interaction	13	2.68E-08
hsa04510	Focal adhesion	16	2.47E-06
hsa04151	PI3K-Akt signaling pathway	16	9.10E-04
hsa05146	Amoebiasis	8	0.002416
hsa04975	Fat digestion and absorption	5	0.004522
hsa05222	Small cell lung cancer	6	0.016089
hsa00561	Glycerolipid metabolism	5	0.018119
hsa04610	Complement and coagulation cascades	5	0.031937
hsa05200	Pathways in cancer	13	0.040692

### Establishment and analysis of the PPI network

**Figure 3:**
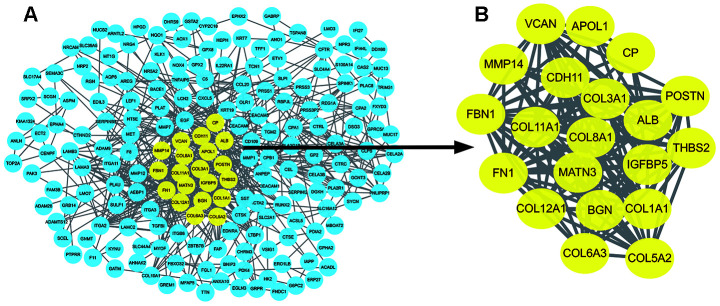
**Identification of hub genes in protein-protein interaction (PPI) network.** (**A**) The PPI network of significant DEGs was constructed using Cytoscape. (**B**) The most significant module selected from the PPI network using the plug-in MCODE of Cytoscape. MCODE :Molecular Complex Detection.

The PPI network of DEGs was constructed using the STRING online database (Figure 3A). The most significant nodes were identified using Cytotype MCODE. 20 central nodes were extracted (Figure 3B) and were used for subsequent analyses.

### Expression validation and survival analysis of core genes

**Figure 4:**
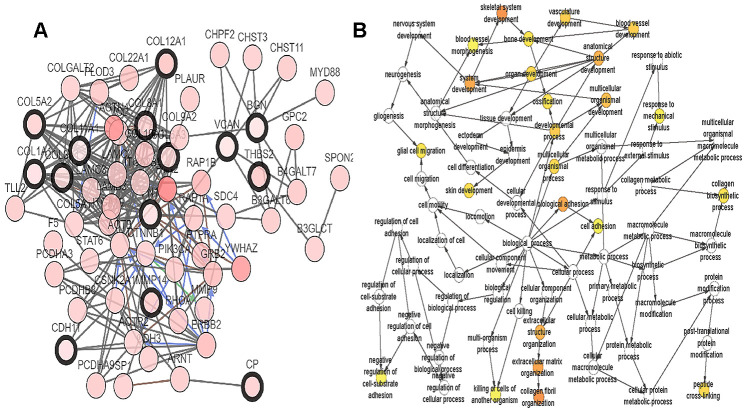
**Interaction network and biological process analysis of the hub genes.** (**A**) Hub genes and their co-expression genes were analyzed using cBioPortal. Nodes with bold black outline represent hub genes. Nodes with thin black outline represent the co-expression genes. (**B**) The biological process analysis of hub genes was constructed using BiNGO. The color depth of nodes refers to the corrected P-value of ontologies. The size of nodes refers to the numbers of genes that are involved in the ontologies. P<0.01 was considered statistically significant.

**Figure 5:**
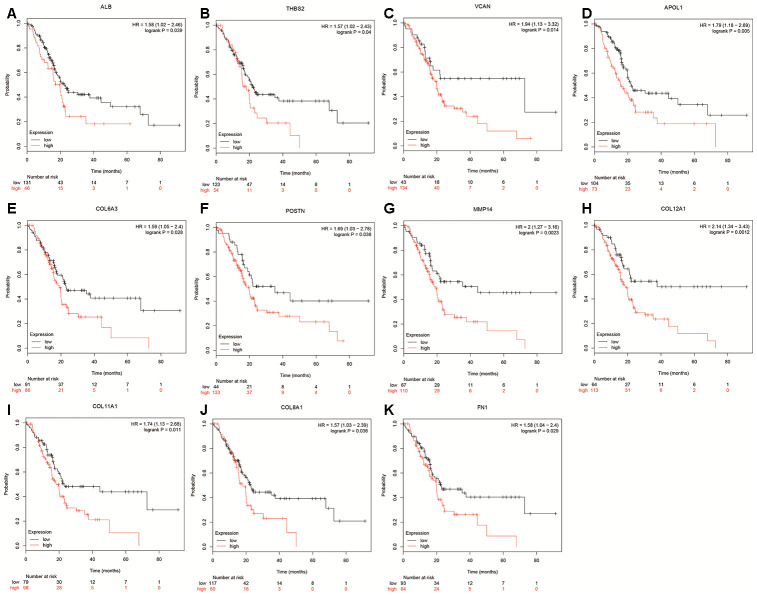
**Overall survival of hub genes were performed using Kaplan-Meier plotter databases.** (**A**) Prognostic value of ALB in pancreatic cancer. (**B**) Prognostic value of THBS2 in pancreatic cancer. (**C**) Prognostic value of VCAN in pancreatic cancer. (**D**) Prognostic value of APOL1 in pancreatic cancer. (**E**) Prognostic value of COL6A3 in pancreatic cancer. (**F**) Prognostic value of POSTN in pancreatic cancer. (**G**) Prognostic value of MMP14 in pancreatic cancer. (**H**) Prognostic value of COL12A1 in pancreatic cancer. (**I**) Prognostic value of COL11A1 in pancreatic cancer. (**J**) Prognostic value of COL8A1 in pancreatic cancer. (**K**) Prognostic value of FN1 in pancreatic cancer. “*”represents “P-value < 0.05”. P<0.05 was considered statistically significant.

**Figure 6:**
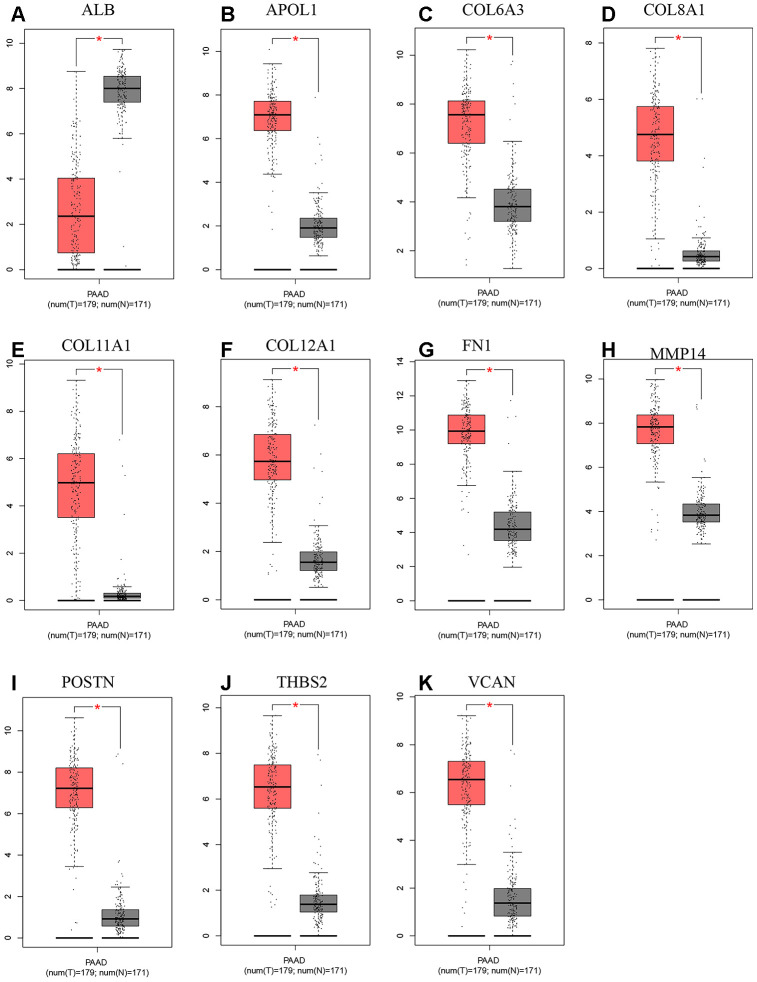
**Expression level between tumor and normal tissues of hub genes in GEPIA.** (**A**) Expression levels of ALB in pancreatic cancer. (**B**) Expression levels of APOL1 in pancreatic cancer. (**C**) Expression levels of COL6A3 in pancreatic cancer. (**D**) Expression levels of COL8A1 in pancreatic cancer. (**E**) Expression levels of COL11A1 in pancreatic cancer. (**F**) Expression levels of COL12A1 in pancreatic cancer. (**G**) Expression levels of FN1 in pancreatic cancer. (**H**) Expression levels of MMP14 in pancreatic cancer. (**I**) Expression levels of POSTN in pancreatic cancer. (**J**) Expression levels of THBS2 in pancreatic cancer. (**K**) Expression levels of VCAN in pancreatic cancer. “*”represents “P-value < 0.05”. P<0.05 was considered statistically significant. Y axis indicates relative expression value, log2(TPM+1). TPM=Transcript per million.

**Figure 7:**
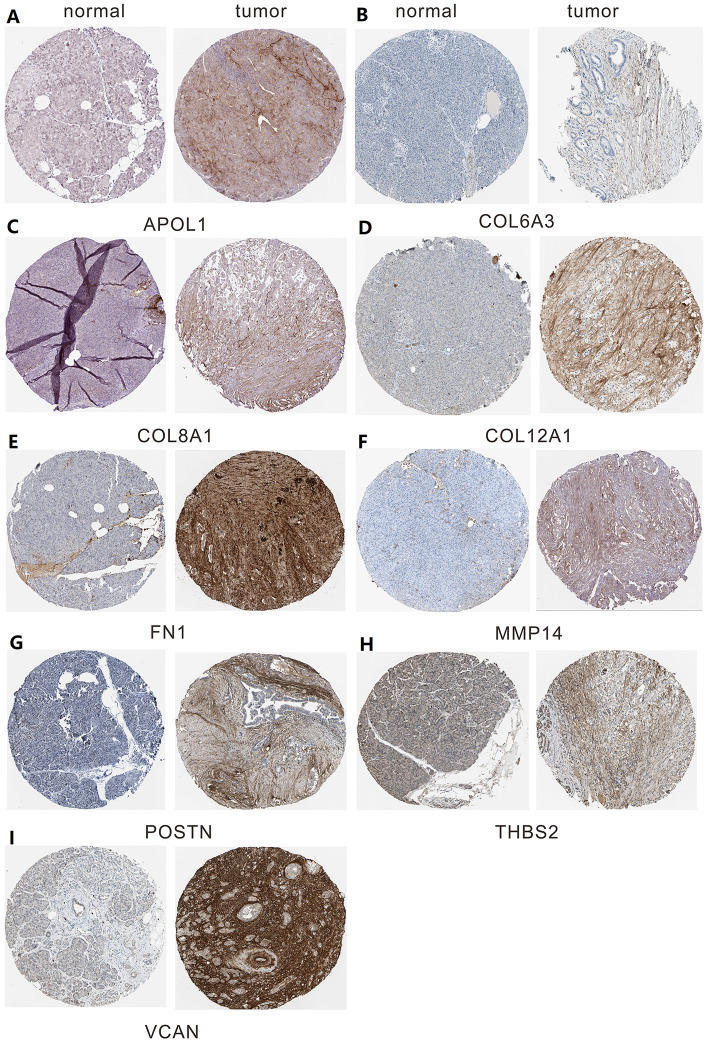
**The protein expression level of hub genes in PDAC tissues and normal tissues was analyzed using immunohistochemical staining from Human Protein Atlas database.** (**A**) Expression levels of APOL1 in pancreatic cancer. (**B**) Expression levels of COL6A3 in pancreatic cancer. (**C**) Expression levels of COL8A1 in pancreatic cancer. (**D**) Expression levels of COL12A1 in pancreatic cancer. (**E**) Expression levels of FN1 in pancreatic cancer. (**F**) Expression levels of MMP14 in pancreatic cancer. (**G**) Expression levels of POSTN in pancreatic cancer. (**H**) Expression levels of THBS2 in pancreatic cancer. (**I**) Expression levels of VCAN in pancreatic cancer.

A total of 20 genes were identified as hub genes by Cytotype MCODE as indicated above. We then used the cBioPortal online platform to analyze the network of the hub genes and their co-expression genes (Figure 4A). The biological process analysis of the core genes was displayed in Figure 4B. The overall survival analysis of the core genes was performed using the Kaplan-Meier plotter (Figure 5). It was found that 11 genes were associated with worse overall survival among the included core genes, including ALB, THBS2, VCAN, APOL1, COL6A3, POSTN, MMP14, COL12A1, COL11A1, COL8A1, and FN1. Then, GEPIA was used to dig up the 11 core genes expression level between cancer patients and normal people. Most of the extracted genes displayed high expression while the ALB gene had a low level in PDAC tissue samples compared to normal pancreatic samples (Figure 6). Finally, we re-analyzed the protein expressions of these 11 genes associated with overall survival on the Human Protein Atlas database (Figure 7).

### hsa-miR-26b-5p-COL12A1 axis is identified as a potential pathway linked to pancreatic carcinoma

**Figure 8:**
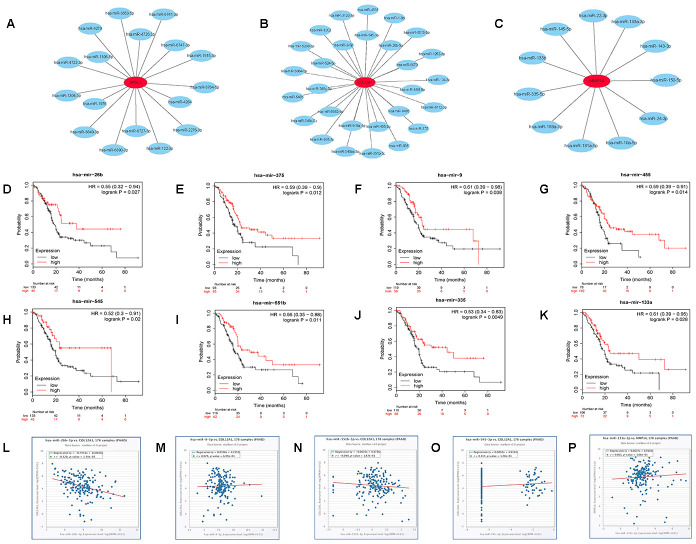
**Identification of upstream potential miRNAs of APOL1, COL12A1 and MMP14 by combination of miRNA prediction, survival analysis and correlation analysis.** (**A**) The miRNA- APOL1 network established by Cytoscape. (**B**) The miRNA- COL12A1 network established by Cytoscape. (**C**) The miRNA-MMP14 network established by Cytoscape. (**D**) Prognostic value of has- miR-26b-5p in pancreatic cancer. (**E**) Prognostic value of has- miR-375 in pancreatic cancer. (**F**) Prognostic value of has-miR-9-5p in pancreatic cancer. (**G**) Prognostic value of has- miR-455-3p in pancreatic cancer. (**H**) Prognostic value of has- miR-545-3p in pancreatic cancer. (**I**) Prognostic value of has-miR-551b-5p in pancreatic cancer. (**J**) Prognostic value of has- miR-335-5p in pancreatic cancer. (**K**) Prognostic value of has- miR-133a-3p in pancreatic cancer. (**L**) The expression correlation of has- miR-26b-5p and COL12A1 in pancreatic cancer. (**M**) The expression correlation of has-miR-9-5p and COL12A1 in pancreatic cancer. (**N**) The expression correlation of has-miR-551b-5p and COL12A1 in pancreatic cancer. (**O**) The expression correlation of has- miR-545-3p and COL12A1 in pancreatic cancer. (**P**) The expression correlation of has- miR-133a-3p and MMP14 in pancreatic cancer.

The prognostic values of the 11 hub genes were further determined using GEPIA to improve accuracy. It was found that the increased expression of the 3 hub genes (COL12A1 APOL1 MMP14) indicated poor prognosis, which was consistent with the survival analysis above. The three genes, COL12A1 APOL1 and MMP14, may be vital in mediating the progression of pancreatic cancer. Subsequently, we predicted the upstream miRNAs of the 3 key genes using miRTarBase, which is an experimentally validated microRNA-target gene interaction database. Finally, 28, 17, and 11 miRNAs were identified to potentially regulate COL12A1, APOL1, and MMP14 expression (Figure 8A–8C and Supplementary Table 1). Given the classical inverse relationship between miRNA and target gene, upstream miRNAs of the 3 genes should theoretically be tumor-suppressive miRNAs, indicating a favorable prognosis. The prognostic values of these predicted miRNAs were then evaluated using the Kaplan-Meier plotter database. As shown in Figure 8D–8K, 8 key miRNAs(miR-375, miR-9-5p, miR-26b-5p, miR-455-3p, miR-545-3p, miR-551b-5p, miR-335-5p and miR-133a-3p) among all the predicted miRNAs showed favorable overall survival. The expression correlation analysis was further assessed. Figure 8L–8P indicated that only hsa-miR-26b-5p was significantly negatively correlated with COL12A1 in pancreatic carcinoma. Hence, the hsa-miR-26b-5p-COL12A1 axis may be a potential pathway in mediating cancer progression of PDAC.

### Upstream potential pseudogenes and lncRNAs of hsa-miR-26b-5p

**Figure 9:**
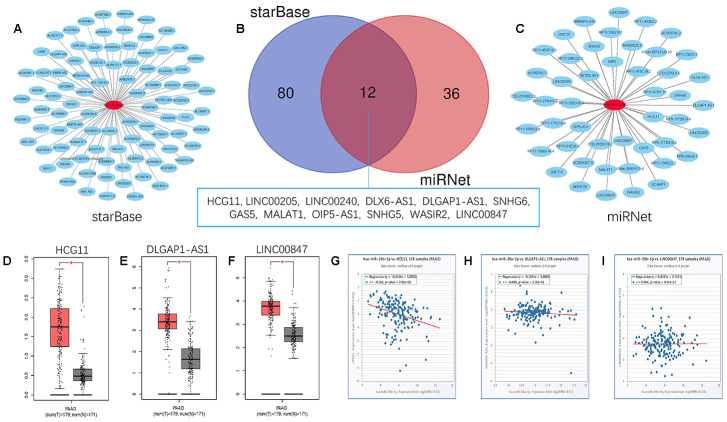
**Screening upstream potential lncRNAs of has- miR-26b-5p in pancreatic cancer.** (**A**) The potential lncRNAs of has- miR-26b-5p predicted by starBase database. (**B**) 12 intersected lncRNAs (LINC00205 LINC00240 DLX6-AS1 DLGAP1-AS1 HCG11 SNHG6 GAS5 MALAT1 OIP5-AS1 SNHG5 WASIR2 LINC00847) from starBase and miRNet databases. (**C**) The potential lncRNAs of has- miR-26b-5p predicted by miRNet database. (**D**) The expression levels of HCG11 in pancreatic cancer (**E**) The expression levels of DLGAP1-AS1 in pancreatic cancer (**F**) The expression levels of LINC00847 in pancreatic cancer. (**G**) The expression correlation of has- miR-26b-5p and HCG11 in pancreatic cancer. (**H**) The expression correlation of has-miR-26b-5p and DLGAP1-AS1 in pancreatic cancer. (**I**) The expression correlation of has-miR-26b-5p and LINC00847 in pancreatic cancer. “*” represents “P-value < 0.05”.

**Figure 10:**
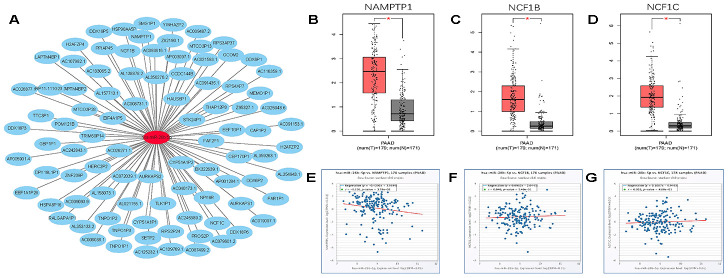
**Screening upstream potential pseudogenes of has- miR-26b-5p in pancreatic cancer.** (**A**) The pseudogenes- has-miR-26b-5p network constructed by Cytoscape. (**B**) The expression levels of NAMPTP1 in pancreatic cancer. (**C**) The expression levels of NCF1B in pancreatic cancer. (**D**) The expression levels of NCF1C in pancreatic cancer. (**E**) The expression correlation of has- miR-26b-5p and NAMPTP1 in pancreatic cancer. (**F**) The expression correlation of has-miR-26b-5p and NCF1B in pancreatic cancer. (**G**) The expression correlation of has- miR-26b-5p and NCF1C in pancreatic cancer. “*” represents “P-value < 0.05”.

**Figure 11:**
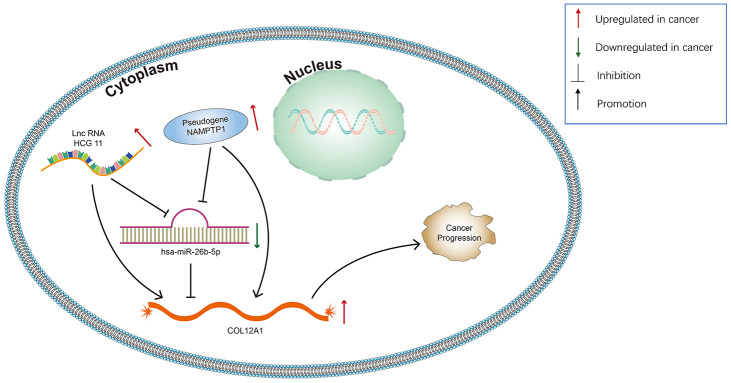
**Model of the pseudogene/lncRNA-has-miR-26b-5p-COL12A1 network and its expression and potential roles in pancreatic cancer progression.**

Growing evidence suggests that pseudogenes and lncRNA may function as ceRNAs to interact with mRNA by competing for shared miRNA. Base on this theory, we predicted the upstream potential lncRNAs that could potentially bind to hsa-miR-26b-5p using starBase and miRNet. 92 and 48 upstream lncRNAs were found in starBase and miRNet as presented in Figure 9A and 9C, respectively. The detailed lncRNAs were listed in Supplementary Table 2. 12 key lncRNAs (LINC00205 LINC00240 DLX6-AS1 DLGAP1-AS1 HCG11 SNHG6 GAS5 MALAT1 OIP5-AS1 SNHG5 WASIR2 LINC00847) were extracted by intersection of the two databases (Figure 9B). Based on ceRNA mechanism, these lncRNAs should be negatively correlated with miRNA in PDAC. Therefore, we analyzed these lncRNAs expression in pancreatic carcinoma using the GEPIA database. Three lncRNAs (DLGAP1-AS1 HCG11 LINC00847) were significantly upregulated in tumor tissues compared to normal controls (Figure 9D–9F). Expression correlation analysis revealed that only HCG11 was significantly negatively correlated with hsa-miR-26b-5p (Figure 9G–9I). A total of 92 pseudogenes that could potentially bind to hsa-miR-26b-5p were also predicted using starBase (Figure 10A and Supplementary Table 3). Only 3 pseudogenes (NAMPTP1 NCF1B NCF1C) were remarkably upregulated in pancreatic cancer samples (Figure 10B–10D) compared to normal controls. Correlation analysis demonstrated that NAMPTP1 exhibited negative relation with hsa-miR-26b-5p (Figure 10E–10G). Based on the ceRNA hypothesis, a novel HCG11/NAMPTP1-miR-26b-5p-COL12A1 triple sub-network was constructed, which showed that overexpressed lncRNAs/pseudogenes mediate downregulation of hsa-miR-26b-5p leading to increased expression of COL12A1 in the progression of pancreatic cancer (Figure 11).

## DISCUSSION

Pancreatic carcinoma is one of the most common gastrointestinal malignancies globally, with no significant clinical signs and symptoms in the initial stages, which results in a poor prognosis [13]. A better understanding of the molecular mechanisms of pancreatic cancer could facilitate earlier diagnosis and the development of more effective treatments [14]. Recent studies have shown that ncRNAs, including miRNAs, lncRNAs and pseudogenes, play vital roles in the initiation and progression of various cancers [15–17]. Wang et al. suggested that the lncRNA MIR210HG promotes cell proliferation and invasion by regulating the MIR210HG/miR-503-5p/TRAF4 axis in cervical cancer [18]. Yang et al. demonstrated that the upregulation of lncRNA LINC00460 facilitates gastric cancer progression via epigenetic silencing of CCNG2 by EZH2/LSD1 and indicates poor outcome [19]. Zhang et al. found that lncRNA LINC00160 functions as a decoy of microRNA-132 and mediates autophagy and drug resistance in hepatocellular carcinoma by inhibiting PIK3R3 [20]. Regarding pseudogenes, the downregulation of transcribed pseudogene RPSAP52 enhances the oncofetal HMGA2-IGF2BP2-RAS axis through impairing the balance between the oncogene LIN28B and tumor suppressor let-7 in human cancers [21]. Kong et al. suggested that pseudogene, PDIA3P1, may promote proliferation and inhibit apoptosis of liver cancer cells by inhibiting the p53 pathway [22]. Many positive pancreatic cancer studies have also been reported. Ren et al. indicated that lncRNA-PLACT1 sustains activation of NF-κB pathway through a positive feedback loop with IκBα/E2F1 axis and promotes proliferation, invasion, and metastasis in pancreatic cancer [23]. Feng et al. found that lncRNA NEAT1 facilitates pancreatic cancer growth and metastasis through stabilizing ELF3 mRNA [24]. However, a comprehensive analysis of ceRNAs in pancreatic cancer is not enough. To the best of our knowledge, this is the first bioinformatics study to introduce pseudogenes into the investigation of specific ceRNA network in pancreatic cancer. Inspiringly, a novel lncRNAs/pseudogenes-miRNA-mRNA triple regulatory network was constructed in our study, and each component may possess a vital role in pancreatic cancer.

In the present study, based on comprehensive expression analysis and survival analysis, three genes (APOL1, MMP14, and COL12A1) were identified as key genes, which may be associated with the progression of PDAC. It was found that these genes were not only significantly increased in pancreatic cancer samples but also that their upregulation was linked to poor prognosis of patients with PDAC. Currently, many studies have demonstrated that these core genes may serve as oncogenes or promising cancer biomarkers. For instance, the activation of apolipoprotein L1 (APOL1) is involved in HSF-1 mediated autophagic cell death in the treatment of colorectal carcinoma using Vitexin [25]. Liu et al. suggested that APOL1 is a prognostic marker in patients with pancreatic cancer using mass spectrometry (MS)- intensive methods [26]. Li et al. showed that the re-expression of MMP14 in hepatocellular carcinoma partially reversed the effect of miR-150-5p by inhibiting cell invasion [27]. Hideaki et al. also reported that inhibition of MMP14 could significantly enhance the anti-invasive effect in PDAC [28]. Kasurinen et al. found that high MMP14 expression predicted worse survival in gastric cancer [29]. Xiang et al. confirmed that COL12A1 could promote gastric cancer metastasis and its level was upregulated in tumors with microsatellite [30]. These studies combined with our current analytic conclusions of expression and survival analysis suggested that APOL1, MMP14, and COL12A1 may be key oncogenes in the progression of pancreatic cancer.

Recent studies suggest that miRNA plays an important role in the initiation and progression of cancer. Therefore, we prepared to seek those miRNAs targeting APOL1, MMP14, and COL12A1. There were 17 potential miRNAs predicted for APOL1 using online miRTarBase databases, 11 for MMP14, and 28 for COL12A1. According to the classical inverse relationship between miRNA and target gene, these miRNAs of 3 upregulated key genes should be tumor-suppressive in pancreatic cancer. After performing survival analysis, eight miRNA-mRNA pairs (miR-375-COL12A1, miR-9-5p-COL12A1, miR-26b-5p-COL12A1, miR-455-3p-COL12A1, miR-545-3p-COL12A1, miR-551b-5p-COL12A1, miR-335-5p-MMP14 and miR-133a-3p-MMP14) functioned as positive prognostic biomarkers in PDAC and were chosen for expression correlation analysis. Correlation analysis showed that only hsa-miR-26b-5p-COL12A1 displayed a significant negative relationship. Numerous studies report that hsa- miR-26b-5p is a key factor in the development of cancers. For example, overexpression of has-miR-26b-5p is associated with decreased cellular proliferation and invasion in hepatocellular carcinoma [31, 32]. Wilke et al. indicated that has-miR-26b-5p acted as a tumor suppressor in radiation-associated breast cancer by inhibiting TRPS1 [33]. Miyamoto et al. found that tumor-suppressive miR-26a-5p inhibits cell aggressiveness by regulating PLOD2 in bladder cancer [34]. These findings partially supported our results. Hence, the hsa-miR-26b-5p-COL12A1 axis may be a potential pathway in regulating the progression of pancreatic cancer.

lncRNAs and pseudogenes could serve as ceRNA and are involved in tumor development by competitively binding to common miRNAs [35]. The upstream lncRNAs of the hsa-miR-26b-5p-COL12A1 axis were first obtained by starBase and miRNet databases. By combining two database results, 12 lncRNAs (LINC00205 LINC00240 DLX6-AS1 DLGAP1-AS1 HCG11 SNHG6 GAS5 MALAT1 OIP5-AS1 SNHG5 WASIR2 LINC00847) were extracted. The GEPIA database was then used to analyze the lncRNA expression; 3 lncRNAs (DLGAP1-AS1 HCG11 LINC00847) were found to be upregulated in tumor tissues as compared to controls. Expression correlation analysis demonstrated that only HCG11 was inversely linked to hsa-miR-26b-5p-COL12A1. The role of lncRNA HCG11 in functioning as oncogenes in some cancers has been investigated. Zhang et al. suggested that lncRNA HCG11 promotes the proliferation and migration of gastric cancer cells through miR-1276/CTNNB1 and the Wnt signaling pathway [36]; Chen et al. showed that lncRNA HCG11 inhibited glioma progression via regulating miR-496/CPEB3 axis and may be a potential therapeutic target [37]; Li et al. found that lncRNA HCG11 accelerates the proliferation and metastasis of hepatocellular carcinoma via miR-26a-5p/ATG12 axis [38]. These reports confirm the reliability of our bioinformatics analysis. Finally, the upstream regulatory pseudogenes of hsa-miR-26b-5p-COL12A1 were also predicted by employing starBase databases. Using GEPIA database, three (NAMPTP1 NCF1B NCF1C) out of 92 predicted pseudogenes were found to be remarkably upregulated in cancer samples. Expression correlation analysis further demonstrated that NAMPTP1 has a negative correlation with miR-26b-5p, indicating that it may play vital roles in regulating hsa-miR-26b-5p-COL12A axis in the progression of pancreatic carcinoma. Notably, there are no studies about the potential molecular regulatory mechanisms of NAMPTP1 in various tumors, which makes it more worthy of further investigation.

## CONCLUSIONS

Based on integrated bioinformatics analysis, a new potential COL12A-miR-26b-5p-HCG11/NAMPTP1 regulatory axis was successfully constructed in pancreatic cancer. To the best of our knowledge, this is the first bioinformatics study to focus on pseudogenes in the investigation of specific ceRNA network in pancreatic cancer. The components of the ceRNA network did not only serve as prognostic biomarkers but also provided key clues for future PDAC molecular mechanistic investigations. However, further studies are required to validate these findings.

## MATERIALS AND METHODS

### Microarray data

Three gene expression datasets (GSE28735, GSE62452 and GSE41368) were downloaded from the GEO database (http://www.ncbi.nlm.nih.gov/geo) using GPL6244 platform (Affymetrix Human Gene 1.0 ST Array). The GSE28735 dataset contained 45 pairs of pancreatic tumors and adjacent non-tumor tissues. The GSE62452 included 69 pancreatic tumors and 61 adjacent non-tumor tissues. The GSE41368 covered 6 PDAC tissue samples and 6 non-cancerous samples.

### Identification of DEGs

We carried out GEO2R (http://www.ncbi.nlm.nih.gov/geo/geo2r) to identify DEGs between pancreatic tumors and non-tumor tissues. The GEO2R is an interactive web tool that aides in the detection of DEGs [39]. In the present study, |logFC|(fold change) >1 and adj. P-value <0.05 were considered statistically significant.

### Gene ontology and KEGG pathway enrichment analysis

The Kyoto Encyclopedia of Genes and Genomes (KEGG) is an online database that can analyze the most significantly enriched pathways of upregulated and downregulated DEGs [40, 41]. The GO is a major bioinformatics tool to annotate genes and analyze the biological process of DEGs, including biological process (BP), cellular component (CC) and molecular function (MF). The Database for Annotation, Visualization and Integrated Discovery (DAVID, https://david.ncifcrf.gov) is an online biological information database and can perform the gene ontology (GO) and KEGG pathway analysis [42].

### PPI network construction and module analysis

The protein-protein interaction (PPI) network was generally evaluated using the Search Tool for the Retrieval of Interacting Genes (STRING; http://string-db.org) (version 10.0) online database [43]. In the current study, a PPI network of DEGs was constructed and visualized using Cytoscape software version 3.5.0 (California, USA), in which the interaction with a combined score >0.4 was identified statistically significant [44]. The plug-in Molecular Complex Detection (MCODE) (version 1.4.2) of Cytoscape was used to identify the most significant module of the PPI network (MCODE scores >5 degree cutoff=2, max. Depth=100, k-core=2, and node score cutoff=0.2) [45].

### Hub genes selection and analysis.

The hub genes were selected through the MCODE Cytoscape app. The cBio Portal (http://www.cbioportal.org) was used to analyze the network of hub genes and their co-expression genes [46, 47]. The Biological Networks Gene Oncology tool (BiNGO) (version 3.0.3) was used to perform and visualize the biological process analysis of hub genes [48]. The overall survival analysis of hub genes was performed using Kaplan-Meier plotter [49]. Meanwhile, the GEPIA website tool was used to validate included DEGs [50]. Finally, the protein expression of hub genes was validated on the Human Protein Atlas database (http://www.proteinatlas.org) by immunohisto-chemistry.

### miRNA prediction

In this study, miRTarbase database was used to predict the upstream miRNAs of key genes, which was experimentally validated by reporter assay, western blot, qPCR, microarray, and next-generation sequencing experiments tool [51].

### starBase and miRNet database analysis

The starBase database is a widely-used platform for studying the ncRNA [11, 52]. it was used to analyze the expression correlation between miRNA and gene or pseudogene/lncRNA. The criteria to identify the significant miRNA-gene/pseudogene pairs was set as R < -0.1 and P-value < 0.05. The starBase database was also used to predict the pseudogenes and lncRNAs that can bind to the included miRNAs. Meanwhile, miRNet database was used to predict the upstream lncRNAs of miRNAs, which is an easy-to-use tool for miRNA-associated studies [53, 54].

## Supplementary Material

Supplementary Tables
