# Supplementary material for: Identification of a new pseudogenes/lncRNAs-hsa-miR-26b-5p-COL12A1 competing endogenous RNA network associated with prognosis of pancreatic cancer using bioinformatics analysis
Source: Aging (Albany NY). 2020 Oct 7;12(19):19107–28. doi: 10.18632/aging.103709 (PMC7732303; doi:10.18632/aging.103709)
Supplement: Supplementary Tables [file aging-12-103709-s001..pdf]

SUPPLEMENTARY TABLES

Supplementary Table 1. The miRNA-mRNA pairs predicted by miRTarBase database.

mRNA	miRNA
APOL1	hsa-miR-4279
APOL1	hsa-miR-4284
APOL1	hsa-miR-2276-3p
APOL1	hsa-miR-1976
APOL1	hsa-miR-3653-5p
APOL1	hsa-miR-1304-3p
APOL1	hsa-miR-6741-3p
APOL1	hsa-miR-6747-3p
APOL1	hsa-miR-6727-3p
APOL1	hsa-miR-4722-3p
APOL1	hsa-miR-4726-3p
APOL1	hsa-miR-6764-5p
APOL1	hsa-miR-1915-3p
APOL1	hsa-miR-6840-3p
APOL1	hsa-miR-6890-3p
APOL1	hsa-miR-1306-5p
APOL1	hsa-miR-122-3p
COL12A1	hsa-miR-375
COL12A1	hsa-miR-9-5p
COL12A1	hsa-miR-124-3p
COL12A1	hsa-miR-1-3p
COL12A1	hsa-miR-26b-5p
COL12A1	hsa-miR-6516-5p
COL12A1	hsa-miR-455-3p
COL12A1	hsa-miR-524-5p
COL12A1	hsa-miR-520d-5p
COL12A1	hsa-miR-5004-5p
COL12A1	hsa-miR-935
COL12A1	hsa-miR-4712-3p
COL12A1	hsa-miR-1252-3p
COL12A1	hsa-miR-548as-3p
COL12A1	hsa-miR-548x-5p
COL12A1	hsa-miR-548g-5p
COL12A1	hsa-miR-548f-5p
COL12A1	hsa-miR-548aj-5p
COL12A1	hsa-miR-605-3p
COL12A1	hsa-miR-545-3p
COL12A1	hsa-miR-5582-5p
COL12A1	hsa-miR-548p
COL12A1	hsa-miR-3120-3p
COL12A1	hsa-miR-8485
COL12A1	hsa-miR-3202
COL12A1	hsa-miR-4533
COL12A1	hsa-miR-6079
COL12A1	hsa-miR-551b-5p
MMP14	hsa-miR-335-5p
MMP14	hsa-miR-145-5p
MMP14	hsa-miR-181a-5p
MMP14	hsa-miR-133a-3p
MMP14	hsa-miR-24-3p
MMP14	hsa-miR-22-3p
MMP14	hsa-miR-10a-5p
MMP14	hsa-miR-193a-3p
MMP14	hsa-miR-150-5p
MMP14	hsa-miR-143-3p
MMP14	hsa-miR-133b

Supplementary Table 2. The potential upstream lncRNAs of hsa-miR-26b-5p predicted by starBase and miRNet databases.

lncRNAs predicted by miRNet	lncRNAs predicted by starBase
NEFL	AL137127.1
SCAMP1	AL359258.3
CWC15	AC239868.1
GAS5	AL358472.3
ZNF718	GAS5
LINC00205	MIR181A1HG
HNRNPU-AS1	AL392172.1
DLX6-AS1	LINC01703
MALAT1	AC104088.1
SNHG5	AC016717.2
HCG11	AC005538.2
SNHG6	THUMPD3-AS1
OIP5-AS1	SH3BP5-AS1
WASIR2	DENND6A-DT
LINC00240	PSMD6-AS2
MIR4720	AC092957.1
AC005082.12	AC098864.1
AC058791.2	AC106744.1
AL589743.1	NNT-AS1
AP000525.9	AC093297.2
CTD-2270L9.5	AC008966.1
CTD-2314B22.3	LINC00847
CTD-2555O16.2	HCG11
DLGAP1-AS1	LINC00240
hsa-mir-6080	SNHG5
LINC00610	AC005082.1
LINC00657	LINC00997
LINC00847	TRG-AS1
RP11-1006G14.4	LINC00174
RP11-105N13.4	AC000120.1
RP11-175O19.4	DLX6-AS1
RP11-276H19.2	AC078846.1
RP11-282O18.3	LINC-PINT
RP11-31E23.1	AP006248.3
RP11-396C23.2	SNHG6
RP11-452F19.3	MSC-AS1
RP11-473I1.10	LINC01111
RP11-478C19.2	AP003476.1
RP11-492E3.2	EBLN3P
RP11-706J10.1	AL513318.2
RP11-738E22.2	AL391056.1
RP11-78O7.2	AL513534.1
RP5-1172N10.3	ENTPD1-AS1
RP5-1172N10.4	RPARP-AS1
RP6-24A23.7	AL139407.1
SETD5-AS1	KCNQ1OT1
XXbac-B461K10.4	AC084117.1
XXbac-BPG32J3.20	NEAT1
	MALAT1
	AP000974.1
	LINC00937
	AC026356.2
	AC144548.1
	AC073857.1
	AC068768.1
	AL122035.1
	AL162171.3
	AL122023.1
	SNHG14
	AC127502.2
	AC013652.1
	OIP5-AS1
	AC023355.1

GABPB1-AS1
AC100827.3
AC105339.2
WASIR2
MMP25-AS1
RRN3P2
AC016876.2
AC087388.1
RP1-178F10.3
AC107983.2
AC015726.1
ARHGAP27P1-BPTFP1-KPNA2P3
AC005332.7
AC005332.6
DLGAP1-AS1
DLGAP1-AS5
ZNF561-AS1
LINC00665
AC022144.1
AC005261.1
AL035661.1
NORAD
LINC00205
DUXAP8
AC016026.1
MIATNB
TUG1
AL022322.1
AL035425.3

Supplementary Table 3. The potential upstream pseudogenes of hsa-miR-26b-5p predicted by starBase database.

Pseudogenes
AL021155.1
GBP1P1
AC242843.1
AC245389.2
LAPTM4BP1
AL157713.1
AL356276.2
AURKAPS1
THAP12P8
AC125232.1
AC093616.1
AC019097.1
YWHAZP2
AC009487.2
LAPTM4BP2
AC026877.1
CYP51A1P1
PROS2P
AC072039.1
HSP90AA5P
MTCO3P28
GCOM2
CEP170P1
TRIM60P14
AC116359.1
AC091435.1
RPS2P24
NPY6R
AL138878.2
RPS4XP7
DDX18P3
DDX6P1

AL354943.1
AL353133.2
EEF1A1P26
DPY19L1P1
HAUS6P1
NCF1B
POM121B
NCF1C
HSPA8P16
EEF1GP1
AC008038.1
H2AFZP2
RP11-111G23.1
AL158073.1
RALGAPA1P1
TLK1P1
NAMPTP1
BX322639.1
CAP1P2
RPS3AP37
TNPO1P1
DDX18P6
FAF2P1
AURKAPS2
H2AFZP4
AP001284.1
AP003097.1
DDX18P5
AC079601.2
PPIAP45
CYP51A1P2
EIF4A1P5
DDX6P2
FAR1P1
SETP2
HERC2P2
AC133065.2
AC040173.1
SMG1P1
AC008731.1
AC009093.9
AC091153.1
TNPO1P2
CCDC144B
AC026271.1
AC107982.1
TNPO1P3
AC087499.2
MTCO3P13
AC025048.6
AC021593.1
AP005901.4
ZNF209P
AC139769.1
MEMO1P1
Z82190.1
TTC3P1
AL359263.1
Z95327.1
STK24P1
